# Metastatic sigmoid adenocarcinoma to the larynx: A case report and updated literature review

**DOI:** 10.1002/ccr3.6942

**Published:** 2023-02-08

**Authors:** Adham A. Aljariri, Abdulqadir J. Nashwan, Rani Hammoud, Bara Wazwaz, Samir Al Hyassat, Hassan Haidar

**Affiliations:** ^1^ Otolaryngology Department Ambulatory Care Center (ACC), Hamad Medical Corporation (HMC) Doha Qatar; ^2^ Nursing Department Hazm Mebaireek General Hospital, Hamad Medical Corporation Doha Qatar; ^3^ Pathology Department Hamad General Hospital (HGH), Hamad Medical Corporation (HMC) Doha Qatar

**Keywords:** colorectal adenocarcinoma, dysphonia, laryngeal metastasis, secondary laryngeal tumors

## Abstract

Metastatic laryngeal cancer is a rare entity, usually indicating an advanced disease once discovered. In this report, we are describing a case of a 60‐year‐old male patient with stage IV colorectal cancer (CRC), who presented to our clinic with dysphonia; further workups showed metastatic CRC.

## Graphical abstract

To avoid life‐threatening effects such as airway obstruction and laryngeal symptoms such as dysphonia should be assessed promptly and treated accordingly in cancer patients. As secondary laryngeal cancers are becoming more common and are being reported more often in the literature.

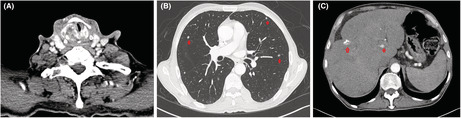

## 1 INTRODUCTION

Colorectal cancer (CRC) is the world's third most common cancer.^1^ The incidence of CRC varies between high‐ and low‐income countries. This variance is due to lifestyle differences and screening programs for premalignant lesions. The presentation of colorectal cancer varies depending on the involved location across the colon. More than 50% of patients die from CRC. Metastasis is usually present in 20% of patients at the time of diagnosis.^2^, ^3^, ^4^, ^5^, ^6^

Local extension of hypo‐pharyngeal and thyroid tumors to the larynx is common. Metastatic laryngeal involvement remains uncommon, accounting for less than 1 percent of all laryngeal malignancies. In 1988, Fertile et al. reported that skin melanoma and renal cell carcinoma as the most common primary cancers to metastasize to the larynx. Since 1988, 13 laryngeal metastases from colorectal cancer have been reported. Other cases are metastasis from the lung, bone, breast, thyroid, liver, and female genital tract.^3^, ^5^

Dysphonia is a term used to describe any impairment or change in the voice. It is caused by irregular vocal muscle oscillation, underlying muscle tension or dysfunction, incomplete closure of the glottis during vocalization, and pressure effect excreted on the vocal folds by a mass or tumor.^4^

## 2 CASE PRESENTATION

A 60‐year‐old Caucasian male patient, known case of Diabetes Mellitus (DM) type II and obstructive sleep apnea, on oral hypoglycemic agents and continuous positive airway pressure (CPAP), respectively, with no previous surgical interventions, was diagnosed with moderately differentiated sigmoid adenocarcinoma Stage IV (with distant metastasis to liver and lung at the time of presentation). The patient was treated with definitive chemotherapy by (FOLFOX/Panitumumab regimen and he completed 12 cycles followed by maintenance therapy with 5FU/Panitumumab).

**FIGURE 1:**
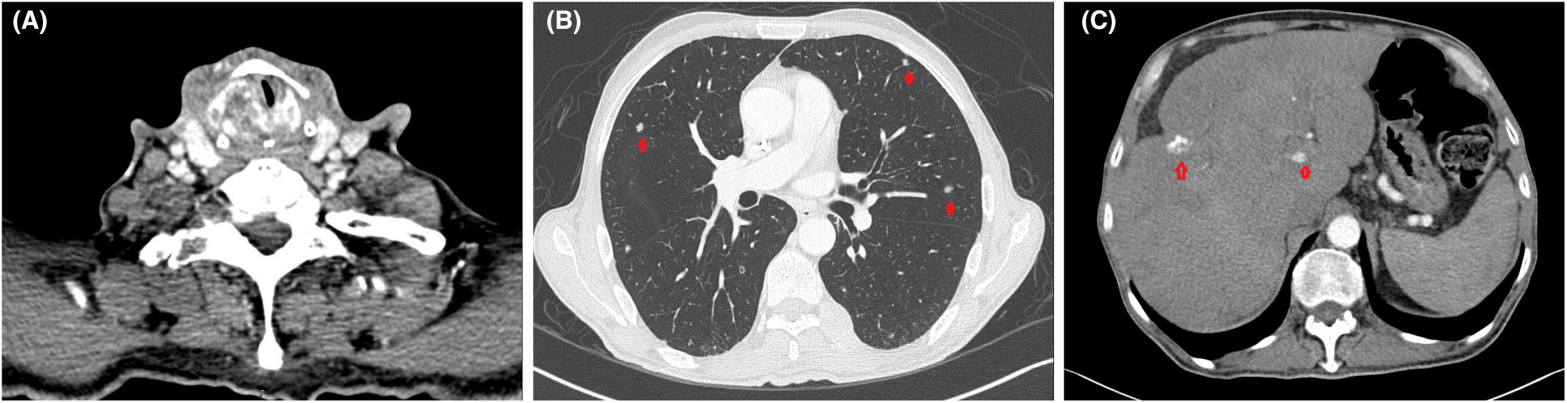
Neck, Chest, and Abdomen CT with contrast showing (A) Right‐sided laryngeal mass involving and destroying the laryngeal cartilages with luminal narrowing and extra laryngeal extension, (B) widely scattered variable‐sized (4–10 mm) pulmonary metastatic nodules, (C) Demonstration of multiple extensive hepatic metastatic involvement).

Subsequently, 18 months after the primary cancer diagnosis, the patient developed dysphonia for 2 weeks, without compressive respiratory symptoms or swallowing difficulties, and thus was referred to the ENT outpatient clinic. Physical examination of the oral cavity, throat, nose, and ears was unremarkable. Examination of the neck revealed no palpable lymphadenopathy. Fiber‐optic examination revealed a paralyzed right vocal cord with a left vocal cord compensation; no masses or lesions were noted along the upper aerodigestive tract. The computerized tomography (CT) scan with contrast of the neck, thorax, and abdomen is displayed in Figure 1, which confirms the presence of the previously recognized distant lung and liver metastasis.

## 3 RADIOLOGY

A contrast‐enhanced neck CT scan (A) demonstrates a destructive right laryngeal mass that is destroying the cartilage and causing luminal narrowing with extra laryngeal extension (Figure 1), Chest and abdomen CT (B, C) with intravenous contrast demonstrates multiple lung nodules of varying sizes (red arrows), along with multiple liver lesions largest measuring (yellow circle).

## 4 PATHOLOGY

**FIGURE 2:**
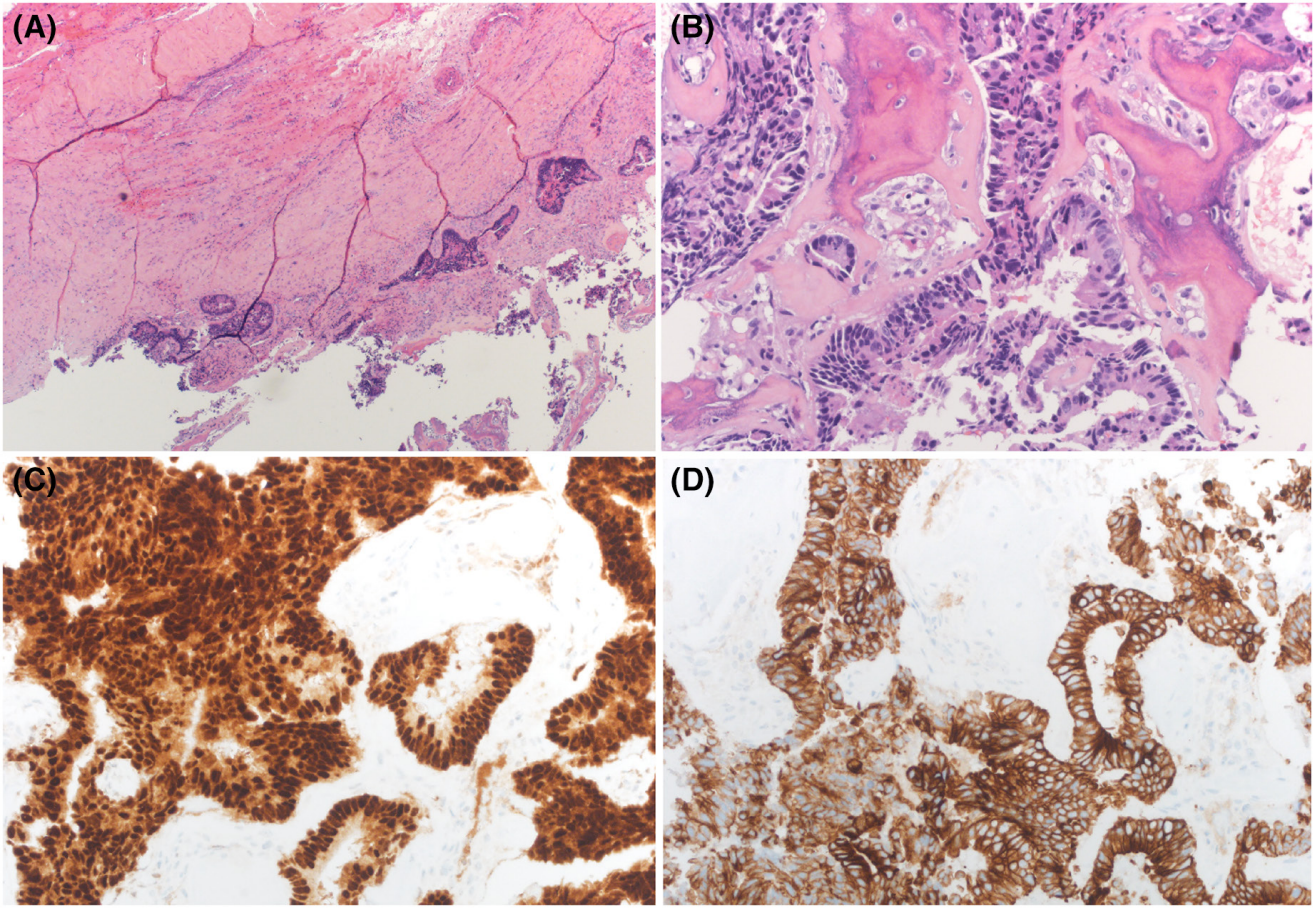
Findings of (A) H&E × 40. (B) H&E × 200. (C) CDX2, and (D) CK20.

Open surgical biopsy (transcervical approach) was obtained from the right cricoid cartilage and histopathology (Figure 2) confirmed the metastatic sigmoid adenocarcinoma of the right cricoid cartilage. (A) Fibrous tissue infiltrated by adenocarcinoma, (B) H&E ×200. Bone infiltrated by adenocarcinoma, (C) Immunohistochemical stain CDX2, and (D) Immunohistochemical stain with CK20. Through the disease course, the patient presented to the emergency department (ED) with complications of stridor and respiratory distress which were promptly managed by ENT via an awake tracheostomy. Later, the patient was sent to continue his treatment in palliative care; however, unfortunately, he was meet with his demise 8 months after the laryngeal metastasis diagnosis.

## 5 DISCUSSION

In metastatic laryngeal involvement, transglottic involvement was the most common cause, compromising more than 40% of the reported cases, followed by supraglottic and subglottic in around 30% each and in less than 10% of the cases the true vocal cords were involved. Approximately, 70% of the cases were reported in men, with 59 years being the median age of diagnosis. The initial presentation in more than 60% was dysphonia, and the median time to laryngeal metastasis diagnosis was 3 years.^3^

The treatment modalities for secondary laryngeal metastasis vary depending on the stage of the disease, the number of metastatic focus, and the involvement of other organs. In 2008, a case reported by Therasma et al., in which the laryngeal metastasis was managed with organ preservation surgery as the patient was in remission.^7^ Another case by Marioni et al. was managed with total laryngectomy due to extensive laryngeal involvement but local control was achieved first at the time of laryngeal diagnosis.^8^ Puxeddu et al., Sano et al., and Ta et al. managed their patients with a tracheostomy to protect the airway from local disease advancement.^9^, ^10^, ^11^ In other cases reported, the local control of the disease was achieved by laser excision by Nd‐YAG laser and CO_2_ laser.^5^, ^12^ A summary of laryngeal metastasis secondary to colorectal cancer is shown in Table 1.

**TABLE 1:** Summary of cases of colon cancer from 1987 to 2022.

	Author	Age/Sex	Country	Initial presentation	Involved site	Management	Other involved sites
1990	Cavicchi et al^16^	59/Female	Italy	Dysphonia	Subglottic	Mass excision	Mediastinal lymph nodes
1996	Nicolai et al^12^	69/Male	Italy	Dysphonia	Glottic	Radiation then total laryngectomy	Lung, Pleura, Brain, Adrenal gland
		53/Female		Dyspnea	Subglottic	Local resection with Laser (Nd‐YAG)	Lung, Lymph nodes
		58/Female		Dyspnea	Subglottic	Local resection with Laser (Nd‐YAG)	Lung, Liver
1997	Puxeddu et al^9^	65/Male	Italy	Dysphonia + respiratory distress	Glottic	Tracheostomy + Local palliative irradiation	Liver, Lung
1998	Hilger et al^17^	73/Female	United Kingdom	Biphasic stridor	Glottic	Tracheostomy followed by extended laryngectomy	Cervical Lymph nodes
2005	Sano et al^10^	81/Female	Japan	Dysphonia and Dyspnea	Subglottic	Tracheostomy	Lung, Sacrum, Liver
2006	Marioni et al^8^	78/Female	Italy	Neck swelling	Glottic	Total laryngectomy and thyroidectomy	Lung, Thyroid
2007	Ramanathan et al^18^	51/Male	Malaysia	Dysphonia	Glottic	Palliative	Liver, Pelvis
2011	Ta et al^11^	60/Male	USA	Dyspnea	Subglottic	Tracheostomy + Debulking	Lung
2014	Therasma et al^7^	54/Male	Japan	Dysphonia	Subglottic	Partial laryngectomy	None
2016	Zenga et al^3^	52/Male	USA	Incidental by PET CT	Glottic	Tracheostomy + Palliative Chemoradiation	Brain, Lung, Sacrum
2017	Heyes et al^5^	56/Female	UK	Shortness of breath	Subglottic	Local excision with CO2 Laser	Lung, Liver
2022	Present case	60/Male	Qatar	Dysphonia	Glottic	Tracheostomy	Lung, Liver

Due to unfamiliarity with secondary laryngeal cancers, there is no consensus on the treatment guidelines; treatment options depend on the stage at the time of diagnosis, solitary laryngeal involvement, or the presence of other metastatic focus. However, it is thought that laryngeal cancer is still underreported, as one postmortem study reported by Prescher et al. showed laryngeal involvement in six autopsies out of six patients with prostate cancer. Similarly, Horny and Kaiserling found 10 of 14 patients with hematopoietic malignancy found to have laryngeal metastasis.^13^, ^14^ Incidental laryngeal metastasis without symptoms is also evident, as reported by Xia et al. when a PET/CT (Positron Emission Tomography/Computed Topography) was performed for an elevated AFP (Alfa Fetoprotein) which showed increased uptake in the larynx.^15^

## 6 CONCLUSION

Any laryngeal lesion in patients with malignancy or high‐risk factors for malignancy should be worked out promptly to avoid any delay in diagnosis and management. Although, secondary laryngeal malignancy is rare, micrometastasis and subclinical disease are evident; however, laryngeal cancer metastasis indicates advanced disease and poor prognosis, and the interventions aim to avoid any respiratory distress or direct mortality related to airway obstruction.

## AUTHOR CONTRIBUTIONS

Adham A. Aljariri, Abdulqadir J. Nashwan, Rani Hammoud, Bara Wazwaz, Sameer Alhyassat, and Hasan A. Haider were involved in data collection, literature search, and manuscript preparation. All authors read and approved the final manuscript.

## FUNDING INFORMATION

This study was not funded.

## CONFLICT OF INTEREST STATEMENT

The authors declare that they have no competing interests.

## ETHICAL APPROVAL

The article describes a case report. Therefore, no additional permission from our Ethics Committee was required.

## CONSENT

A written informed consent was obtained from the patient to publish this report in accordance with the journal's patient consent policy.

## Data Availability

All data generated or analyzed during this study are included in this published article.
